# Density dependence forces divergent population growth rates and alters occupancy patterns of a central place foraging Antarctic seabird

**DOI:** 10.1002/ece3.6037

**Published:** 2020-02-20

**Authors:** Colin Southwell, Louise Emmerson

**Affiliations:** ^1^ Australian Antarctic Division Department of the Environment and Energy Kingston Tas. Australia

**Keywords:** Adélie penguin, competition, density dependence, numerical response, occupancy, population regulation, resource availability

## Abstract

Density‐dependent regulation is an important process in spatio‐temporal population dynamics because it can alter the effects of synchronizing processes operating over large spatial scales. Most frequently, populations are regulated by density dependence when higher density leads to reduced individual fitness and population growth, but inverse density dependence can also occur when small populations are subject to higher extinction risks. We investigate whether density‐dependent regulation influences population growth for the Antarctic breeding Adélie penguin *Pygoscelis adeliae*. Understanding the prevalence and nature of density dependence for this species is important because it is considered a sentinel species reflecting the impacts of fisheries and environmental change over large spatial scales in the Southern Ocean, but the presence of density dependence could introduce uncertainty in this role. Using data on population growth and indices of resource availability for seven regional Adélie penguin populations located along the East Antarctic coastline, we find compelling evidence that population growth is constrained at some locations by the amount of breeding habitat available to individuals. Locations with low breeding habitat availability had reduced population growth rates, higher overall occupancy rates, and higher occupancy of steeper slopes that are sparsely occupied or avoided at other locations. Our results are consistent with evolutionary models of avian breeding habitat selection where individuals search for high‐quality nest sites to maximize fitness returns and subsequently occupy poorer habitat as population density increases. Alternate explanations invoking competition for food were not supported by the available evidence, but strong conclusions on food‐related density dependence were constrained by the paucity of food availability data over the large spatial scales of this region. Our study highlights the importance of incorporating nonconstant conditions of species–environment relationships into predictive models of species distributions and population dynamics, and provides guidance for improved monitoring of fisheries and climate change impacts in the Southern Ocean.

## Graphical abstract

Using data on population growth and indices of resource availability for seven regional Adélie penguin populations located along the East Antarctic coastline, we find compelling evidence that population growth is constrained at some locations by the amount of breeding habitat available to individuals. Locations with low breeding habitat availability had reduced population growth rates, higher overall occupancy rates, and higher occupancy of steeper slopes that are sparsely occupied or avoided at other locations. Alternate explanations invoking competition for food were not supported by the available evidence, but strong conclusions on food‐related density dependence are constrained by the paucity of food availability data over the large spatial scales of this region

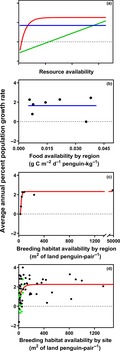

## 1 INTRODUCTION

Density dependence is a central tenet in population ecology where outcomes of interactions between individuals and their environment are governed by the density of individuals (Krebs, 2002). Most frequently, populations experience density dependence when higher density leads to increased competition for resources, higher detection by predators, or greater susceptibility to disease or parasites, with consequent reduction in individual fitness and population growth. There are circumstances, however, where inverse density dependence occurs when small populations have higher extinction risks through genetic inbreeding, demographic stochasticity, or reduced cooperative interactions with conspecifics (Courchamp, Clutton‐Brock, & Grenfell, 1999; Stephens, Sutherland, & Freckleton, 1999). Inverse density dependence can also occur when density is high, for example, by facilitating cooperative vigilance or defense against predators (Pays, Jarman, Loisel, & Gerard, 2007). The net balance between the positive and negative effects of density dependence thus depends on the interplay between the characteristics of a species life‐history, ecology, and environment. An important consequence of local density‐dependent regulation is that it can disrupt the synchronizing effects on population dynamics of processes such as dispersal, environmental fluctuations, and trophic interactions that operate at large spatial scales (Bjørnstad, Ims, & Lambin, 1999; Ranta, Kaitala, Lindström, & Lindén, 1995).

For seabird species, the focal group here, life‐history characteristics including geographically separated or patchy breeding habitat, high coloniality, high philopatry, and central place foraging may result in within‐patch dynamics such as density dependence being a driving or constraining force on population dynamics. Processes that regulate seabird population dynamics can occur on land where they breed or in the ocean where they forage through, for example, habitat availability and competition for food, respectively. Evolutionary models of avian breeding habitat selection predict that when habitat quality is spatially heterogeneous and individuals search for high‐quality habitat to maximize their fitness, individuals will occupy the best nest sites first in a preemptive manner to maximize their fitness returns (the ideal despotic distribution model, Fretwell & Lucas, 1969). According to this model, density dependence will lead to reduced population growth rates as poorer sites are used at higher population densities, even if breeding success at individual nests does not change with density (Gadenne, Cornulier, Eraud, Barbraud, & Barbraud, 2014; Kokko, Harris, & Wanless, 2004). Alternately, positive outcomes from density dependence may occur if higher density of neighboring conspecifics enhances the defense of unattended chicks from predator attack when parents spend time away from the nest to forage (Ashbrook, Wanless, Harris, & Hamer, 2010). At sea, models of seabird foraging dynamics predict that a zone of depleted prey will develop around large breeding colonies which can reduce individual fitness through greater energetic costs in accessing prey and reduced fecundity (Ashmoles halo: Ashmole, 1963; Birt, Birt, Goulet, Cairns, & Montevecchi, 1987; Cairns, 1989; Furness & Birkhead, 1984; Gaston, Ydenberg, & Smith, 2007; Storer, 1952). This can also result in a negative structuring of breeding colonies whereby large colonies are surrounded by small neighboring colonies (Ainley, Ford, Brown, Suryan, & Irons, 2003; Ainley, Nur, & Woehler, 1995).

**Figure 1:**
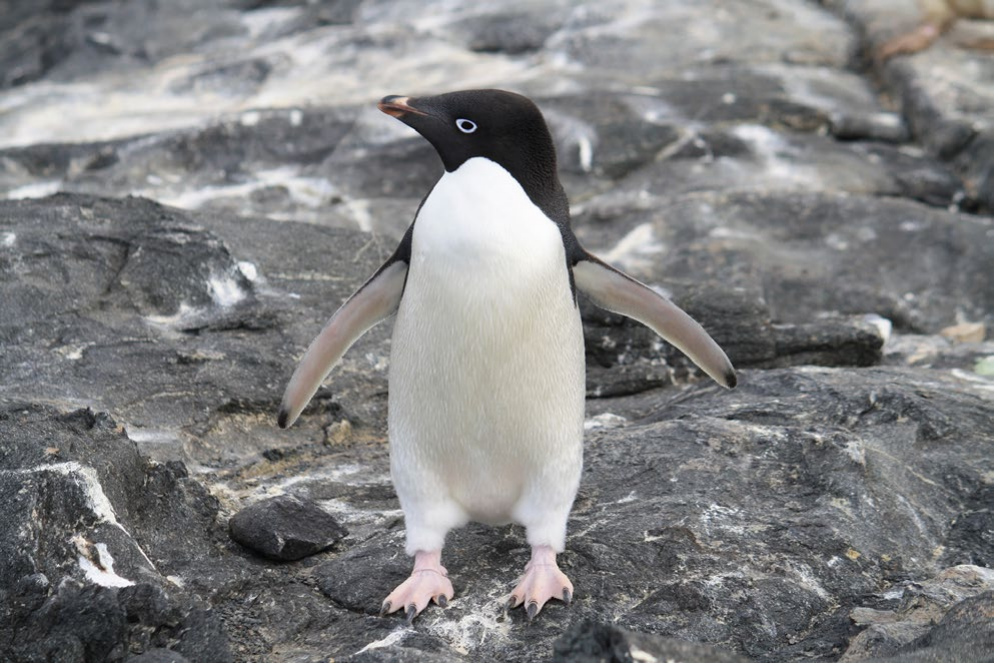
The Adélie penguin

Here, we assess evidence for density‐dependent population regulation in an Antarctic seabird, the Adélie penguin *Pygoscelis adeliae* (Figure 1). Although this species is well studied at locations throughout its circumpolar range, to our knowledge no studies have examined the effect of per capita resource abundance, which for brevity we hereafter term resource availability, on population growth. Understanding density dependence in this species is important because it is widely considered a sentinel species reflecting the impacts of fisheries and environmental change in the Southern Ocean (Agnew, 1997; Ainley, 2002), but the presence of density‐dependent population regulation could dampen population change and thus make it difficult to identify these impacts.

In East Antarctica, Adélie penguins breed on clusters of islands and coastal rock outcrops separated by long stretches of ice‐cliffs and ice‐shelves that are unsuitable for breeding. We use the term “local population” to refer to breeders on an individual island or rock outcrop, and “regional population” to clusters of local populations separated by distances greater than their summer foraging ranges. Breeding occurs during the austral summer from October to March (Ainley, 2002; Emmerson, Pike, & Southwell, 2011), and during the breeding season penguins travel up to 400 km from their breeding sites to forage in pelagic waters of the Southern Ocean (Clarke, Emmerson, & Otahal, 2006; Cottin et al., 2012; Kato, Yoshioka, & Sato, 2009; Kerry et al., 1997; Wienecke et al., 2000). The wide range in regional population sizes, which vary by up to two orders of magnitude (Southwell et al., 2017), and the variable characteristics of their marine and terrestrial environments, provides an ideal opportunity to test whether population growth is constrained or enhanced by density‐dependent resource availability at‐sea or on‐land during the breeding season. Using data on population growth rate and physical and biological attributes of the marine and terrestrial environments of regional Adélie penguin populations along 5,000 km of the East Antarctic coastline, we find compelling evidence that population growth is constrained by resource availability limitations at both regional and local scales.

## 2 MATERIAL AND METHODS

Our investigation of density‐dependent population regulation focuses on seven regional Adélie penguin populations with published long‐term population data. We characterized the general physical and biological attributes of the marine and terrestrial environments used by these populations and used combinations of these attributes to develop specific indices of the availability of food and breeding resources. We used these indices as covariates to model population growth rate in relation to resource availability and test whether there was evidence for density dependence across the range of resource availabilities experienced by the populations. If evidence of density‐dependent regulation was found, we examined additional data on the underlying mechanisms contributing to density dependence. Details of these steps are below.

### 2.1 Population growth rates

**Figure 2:**
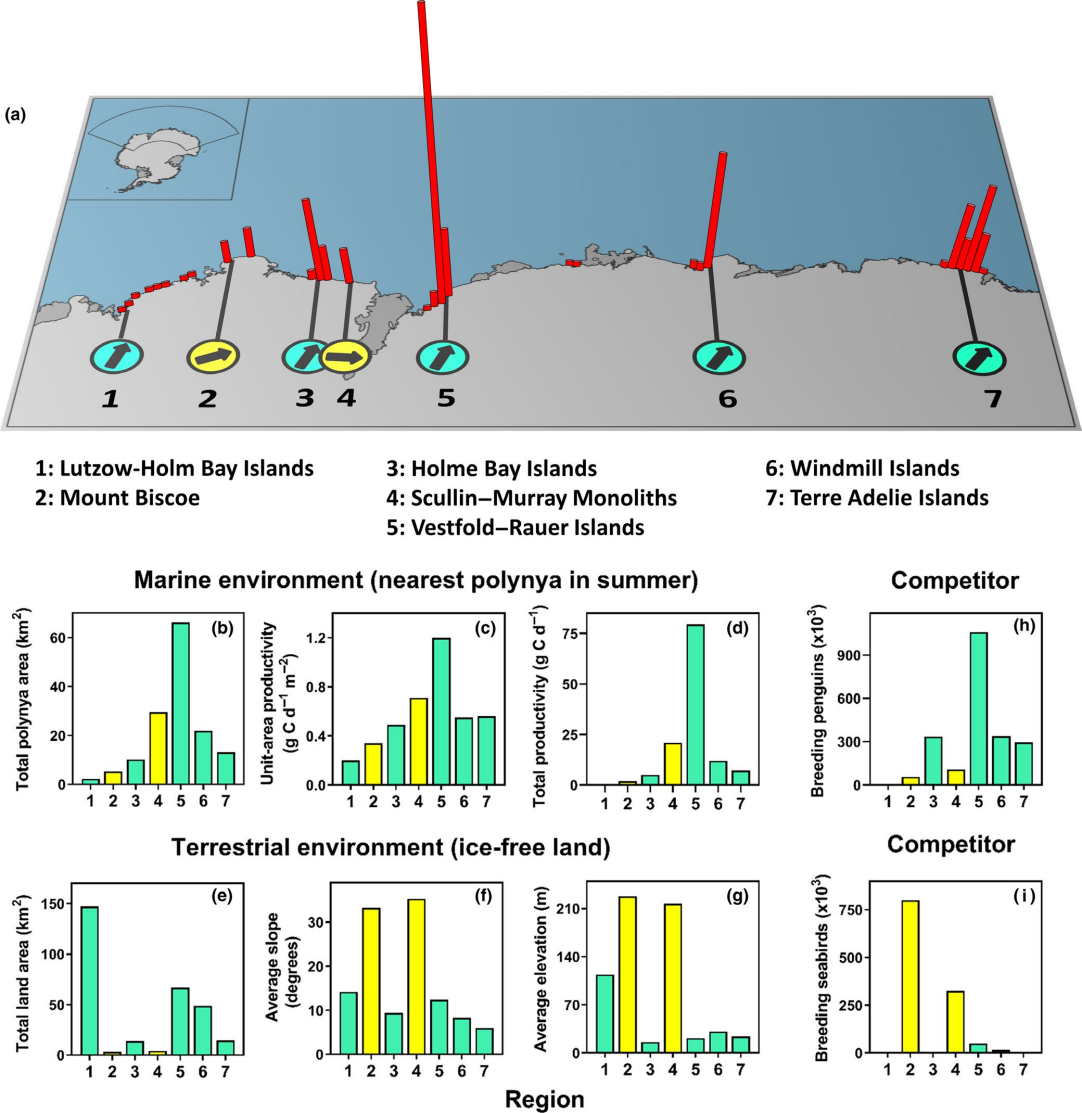
Distribution, population size, population growth rates, and characterization of marine and terrestrial habitats of seven regional Adélie penguin breeding populations along the East Antarctic coastline. (a) Vertical red bars indicate the distribution of breeding populations as the number of breeding‐age penguins within one‐degree increments of longitude, with the largest bar representing approximately one million penguins (from Southwell et al., 2017). Arrows within circles indicate the average annual percent population growth rate over the past three decades: Horizontal is no change, vertical‐up is 3% increase, and vertical‐down is 3% decrease. Numerical values for the arrows are in Appendix S3. (b)–(i) physical and biological features of marine and terrestrial environments of each region. Bars within panels are regions ordered west to east, numbered as in panel (a). Bars and circles are colored green for island archipelago and yellow for mountain nunatak habitats

We collated empirical data on population growth rate at each of the seven regional Adélie penguin populations from population count data presented in recent publications (Lynch & LaRue, 2014; Southwell & Emmerson, 2019; Southwell et al., 2017). We concluded, after careful scrutiny, that the published estimate of population growth at Mount Biscoe (region 2, Figure 2a) was based on an unreliable baseline count (Appendix S1), and instead estimated population growth for this region from published accounts of guano area (Appendix S2). Population growth rate for each region was calculated as the average annual instantaneous rate of change by estimating the slope of the linear regression of the natural logarithm of repeated region‐wide population size or guano area estimates against year. We then converted this metric to the average annual percent rate of change for presentation, as it is more intuitive. The population growth rate estimates are summary metrics of average long‐term rate of change across the span of each time series and should not be taken to imply that change has necessarily been constant over time. Prior to calculating growth rates, count data were standardized to a common metric, the maximum number of occupied nests, using the methods in Southwell et al. (2015) to ensure reliable comparison across time. The standardization process used bootstrap methods to account for uncertainties associated with the timing of population counts, and these uncertainties were propagated through to uncertainties around the estimates of regional population growth rate. The estimates of population growth rate (median and 95 percentile range) are in Appendix S3.

### 2.2 Characterizing marine and terrestrial environments

To characterize the marine environment, we considered attributes of the closest polynya to each regional population. Polynyas are areas within the sea‐ice zone with predictably low sea‐ice cover and high productivity (Arrigo & Van Dijken, 2003; Massom, Harris, Michael, & Potter, 1998; Massom et al., 2013), and can be important foraging grounds for marine predators such as Adélie penguins (Ainley, 2002; Ainley et al., 2010; Arrigo & Van Dijken, 2003; Karnovsky, Ainley, & Lee, 2007; Wilson et al., 2001). We identified the closest polynya to each regional population and collated published data on its area, daily primary productivity per unit area, and total daily primary productivity in the austral summer from Arrigo and Van Dijken (2003). These data had been averaged over five austral summers from 1997/98 to 2001/02, which aligns approximately with the middle of the periods over which regional population growth rates were calculated. We used total primary productivity as a proxy for the abundance of mid‐trophic level biota such as krill and fish that are food resources for Adélie penguins.

To characterize the terrestrial environment of each regional population, we collated data on the total area, average slope, and average elevation of ice‐free land as physical attributes relevant to breeding resources. These data were calculated for all areas of land across the east–west extent of each regional population and were sourced from a database of potential Adélie penguin breeding habitat in East Antarctica (Southwell, Emmerson, Smith, & Bender, 2016) and the ASTER global digital elevation model (Rees, 2012).

Finally, we considered the abundance of Adélie penguins and flying seabirds breeding on land and feeding in adjacent waters as biological attributes relevant to both food and breeding resources (through potential intra‐ and interspecific competition), and compiled estimates of Adélie penguin and flying seabird populations breeding in each region from the published literature.

### 2.3 Indices of resource availability

The amount of food available for individual breeding penguins is a function of the total amount of food present in their foraging range and the level of competition for that food by conspecifics or other species. To derive an index of food availability in each regional penguin population, we divided the total primary productivity in the nearest polynya by the biomass of potential avian competitors breeding in the region. We calculated this index using Adélie penguin biomass only to reflect potential intraspecific competition and also using the combined biomass of all seabird species to reflect intra‐ and interspecific competition. We used biomass rather than population size in the calculation to account for the differing body sizes of penguins and flying seabird species (~5 kg and 600 g, respectively), and therefore their food requirements.

Given the sparsity of ice‐free land in Antarctica (Lee et al., 2017), the most important aspect of Adélie penguin breeding requirements that could limit population growth is the area of breeding habitat (e.g., LaRue et al., 2013). Adélie penguins prefer nest sites that are close to the coast and on gentle to moderate slopes to minimize the energetic costs of accessing them (Ainley, 2002, results and unpublished data). The species is also highly philopatric under all but extreme environmental conditions (Dugger, Ainley, Lyver, Barton, & Ballard, 2010) and exhibits low colonization rates even when populations are increasing (Southwell & Emmerson, 2013). Consequently, we defined potential Adélie penguin breeding habitat as ice‐free land within 500 m of the coast and with slope <45° at currently occupied breeding sites (where a site is an island or rock outcrop bounded by ocean or ice) and calculated an index of breeding habitat availability for each region by (a) calculating the total area of potential breeding habitat in the region, (b) subtracting the area occupied by breeding Adélie penguins in the first breeding season of population data (assuming a nesting density of 1 nest/m^2^) to give the area of unoccupied potential habitat available for population growth, and (c) dividing this area by the Adélie penguin breeding population size in the first breeding season of the population time series to estimate per capita unoccupied potential breeding area. In contrast to the food availability index, we did not consider flying seabirds as potential competitors with Adélie penguins for breeding habitat because their breeding habitat preferences differ substantially (Ainley, 2002; van Franeker, Gavrilo, Mehlum, Veit, & Woehler, 1999).

### 2.4 Density dependence

We used an information‐theoretic approach to assess the level of support for three increasingly complex candidate models (Figure 4a) of density dependence between population growth rate (pgr) and resource availability (*r*):

Candidate 1: density dependence absent across the range of measured resource availabilities, represented by the null model:

pgr=a

where pgr is at a constant level *a* across resource availabilities;

Candidate 2: density dependence present across the full range of measured resource availabilities, represented by the linear model:

pgr=b+cr

where pgr increases linearly with increasing *r* from a minimum level *b* at rate *c*, and

Candidate 3: density dependence present across a partial range of measured resource availabilities, represented by the nonlinear model:

$$\text{pgr}=d+e\left(1-\exp^{-fr}\right)$$

where *d* is the maximum rate of decrease that occurs in the absence of a resource, *e* is a constant describing the difference between *d* and the maximum rate at which a population can increase, and *f* is the demographic efficiency of a population indexing how quickly pgr changes from being negative to positive as a resource increases (Bayliss & Choquenot, 2002). Under this model, pgr increases asymptotically with increasing *r*.

To assess the level of support for the null, linear, and nonlinear models at the regional scale, we fitted each of the three candidate models to regional population growth rate and resource availability data and calculated Akaike's information criterion (AIC) using R (R Core Team, 2016). After correcting AIC values for small sample size (AIC_c_), ∆AIC_c_ values were calculated as the difference between each model's AIC_c_ value and the minimum AIC_c_, and the models were ranked by their ∆AIC_c_ values and Akaike weights. Models with ∆AIC_c_ ≤ 2 were considered to be strongly plausible, 3 ≤ ∆AIC_c_ ≤ 7 considerably less plausible, and ∆AIC_c_ ≥ 7 improbable (Burnham & Anderson, 2002). We also calculated the percent of the null model's deviance explained by linear and nonlinear models. We repeated this process with local (island)‐scale breeding habitat availability data to assess whether there was evidence for density dependence at the local scale. This was only possible for breeding habitat availability because the likely overlap in foraging ranges of penguins from islands in each region would result in little or no differentiation of the data at the site level. A total of 65 local populations that were extant at the start of the time series were included in this analysis. Local population data available to us for this analysis were from four of the seven regions.

### 2.5 Searching for density dependence mechanisms

If pgr showed a relationship with a resource availability covariate, we searched for the mechanisms by which density dependence could occur. In the case of breeding habitat availability, we examined the Adélie penguins' use of terrestrial habitat by investigating the level of occupancy of ice‐free land by breeding penguins in relation to region and slope. This analysis was possible for five of the seven regional populations (regions 2–6; Figure 2a) for which maps of breeding colony boundaries were available. To quantify occupancy, breeding colony boundaries which had been digitally mapped with a hand‐held GPS from the ground or from geo‐referenced vertical aerial photographs were overlaid on a grid of 50 × 50 m plots in a GIS. Each plot was classified as occupied or unoccupied according to whether it overlapped a colony boundary or not, and the average slope in degrees for each plot was calculated from the DEM.

Generalized linear regression models (family = binomial, link function = logit) were used to model the probability of a plot being occupied as a function of region (factor) and slope (continuous variable). First‐ and second‐order slope terms were considered to assess for linearity versus curvilinearity in occupancy–region–slope relationships. An information‐theoretic approach was used to select the most parsimonious models from a set of seven models ranging from a null model of constant occupation to a global model including region, slope, slope^2^, and the interaction of region and slope.

## 3 RESULTS

The seven regional Adélie penguin breeding populations are widely distributed along the East Antarctic coastline, vary in size by over two orders of magnitude, and have population growth rates ranging from −0.01% to 2.45% per annum (Figure 2a, Appendix S3).

### 3.1 Marine and terrestrial environments

The regional populations are characterized by widely varying marine and terrestrial environments (Figure 2b–i). Total primary productivity in the marine environment, for example, varies by 1–2 orders of magnitude (Figure 2d), with the highest level for the polynya closest to the Vestfold – Rauer Islands' population (region 5) due to its large size (Figure 2b) and high productivity per unit area (Figure 2c). Conversely, the polynya closest to the Lützow‐Holm Bay Islands population (region 1) is the smallest, has the lowest productivity per unit area, and hence has the lowest total productivity. Terrestrial environments vary from ocean‐adjoining mountain nunataks at Mount Biscoe and Scullin–Murray Monoliths (regions 2 and 4; Figure 2a) to island archipelagos elsewhere. The terrestrial environment of the two nunatak populations is characterized by smaller areas, steeper slopes, and higher elevations compared with the island archipelago populations (Figure 2e–g). The island archipelago and mountain nunatak habitats support different avifauna assemblages. With the exception of the Lützow‐Holm Bay Islands' population (region 1) which has overall small avian populations, Adélie penguins are more abundant in the island archipelago regions than the mountain nunatak regions (Figure 2h). This reflects both the larger area of land in the archipelagos and the inability of penguins to access the steeper slopes of the mountain nunataks. In contrast, the mountain nunatak habitats tend to have smaller regional penguin populations but support large breeding populations of surface‐nesting flying seabirds, in particular Antarctic petrels *Thalassoica antarctica* which breed on the steep slopes (Figure 2i).

### 3.2 Resource availability indices

**Figure 3:**
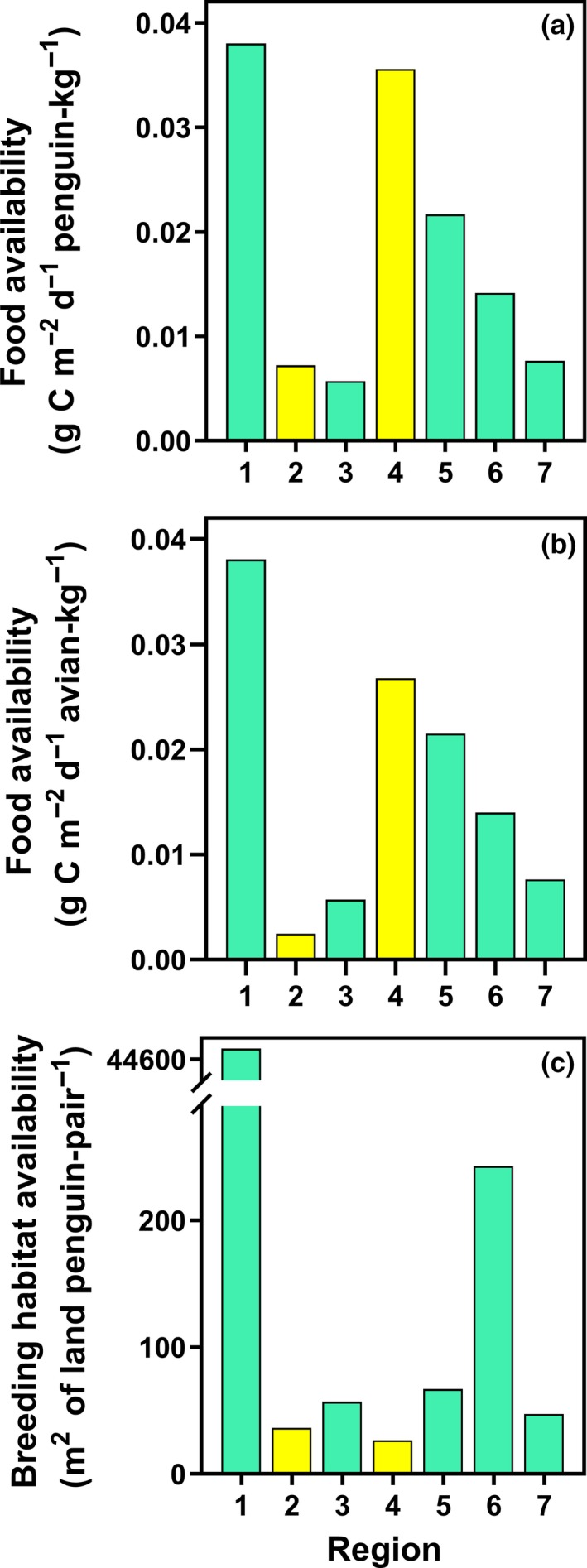
Resource availability at seven regional Adélie penguin populations in East Antarctica. Panels are different resource availability indices. Bars within panels are regional populations ordered from west to east (numbered 1–7) as in Figure 2, and colored green for island archipelago and yellow for mountain nunatak habitats. (a) Food availability allowing for intraspecific competition; (b) food availability allowing for intra‐ and interspecific competition; (c) breeding habitat availability

The pattern of food availability indices across regions is similar irrespective of whether intraspecific competition or both intraspecific competition and interspecific competition are considered. Food availability indices are lower at Mount Biscoe, Holme Bay Islands, and Terre Adélie Islands (regions 2, 3, and 7) than elsewhere (Figure 3a,b). The mountain nunatak populations have lower indices of breeding habitat availability than the island archipelago populations (Figure 3c).

### 3.3 Density dependence

**Figure 4:**
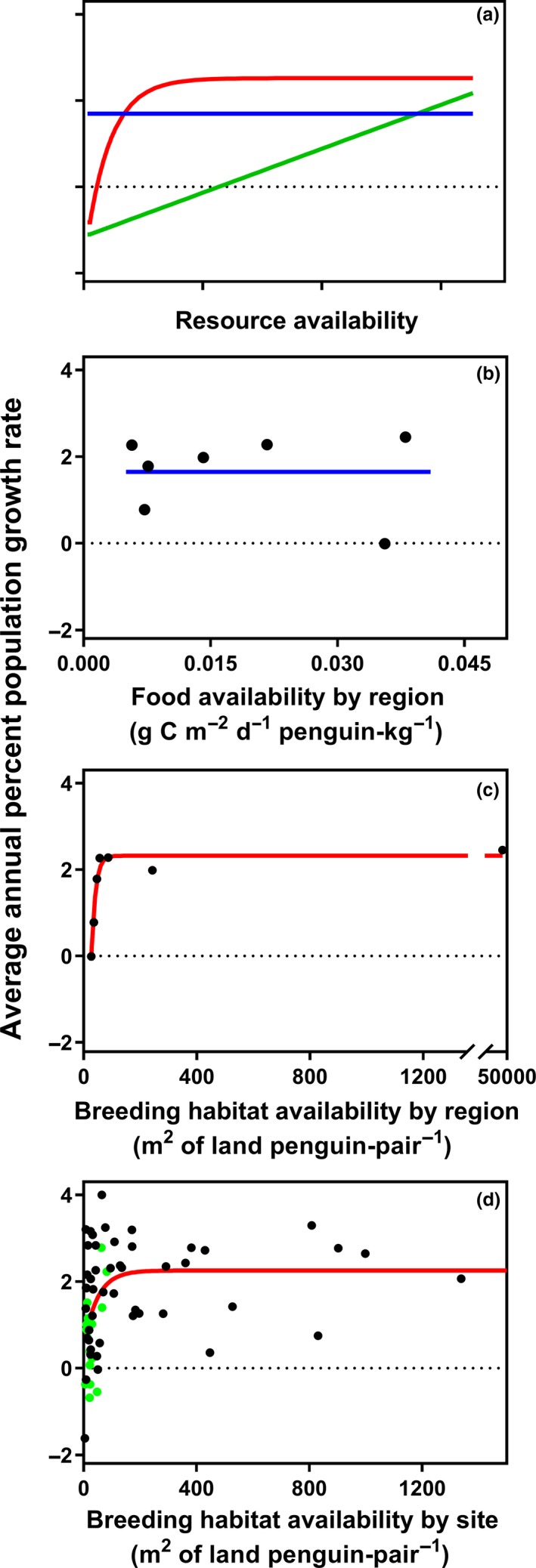
Model selection results. (a) General form of null, linear, and nonlinear candidate models for the relationship between population growth rate and resource availability. (b) and (c) Fit of the most parsimonious models to regional population growth rate and food and breeding habitat availability data. Only one of the food availability indices is shown because data and model fits for the two indices are similar. (d) Fit of the most parsimonious model to local population growth rate and breeding habitat availability data in four regions. Islands with total breeding habitat area ≤0.5 km^2^ shown in green. In all panels, null models are blue, linear models are green, nonlinear models are red, and dashed gray lines indicate zero growth (stable population)

There was strong support for density dependence in regional population growth at low levels of breeding habitat availability, with the nonlinear model having a 99% probability of being the best model in the candidate set and explaining 95% of the null model's deviance (Table 1, Figure 4c). The fitted numerical response model predicts regional‐scale pgr declines below a maximum level of 2.3% per annum when there is <80 m^2^ of unoccupied habitat available per breeding pair, and declines to zero growth (i.e., stable population) at 28 m^2^ of unoccupied habitat per breeding pair. In contrast, we found no evidence in support of density‐dependent population growth at the regional scale in response to food availability. In scenarios of both potential intraspecific competition only and intra‐ and interspecific competition combined, the null model was a more parsimonious fit to the data than the linear model, and nonlinear models failed to converge (Table 1). Visual inspection of pgr and food availability data gave no suggestion that nonlinear models were a suitable fit.

**Table 1:** Model selection results for three candidate models of increasing complexity for density dependence of population growth rate in response to resource availability (null model: absence of density dependence across the measured range of resource availabilities; linear model: presence of density dependence across the full measured range of resource availabilities; nonlinear model: presence of density dependence across part of the measured range of resource availabilities). Models for each resource are ranked in order of Akaike weights (*w_i_*), and those with substantial support (∆AIC_c_ < 2) are shown in bold. *K* is the number of estimated parameters for the model. ‐ Model failed to converge

Spatial scale	Resource	Candidate models for density dependence	*K*	AIC_c_	∆AIC_c_	*w_i_*	% of null model's deviance explained
Regional	Food (allowing for intraspecific competition only)	**Null**	**1**	**22.38**	**0**	**0.88**	**–**
		Linear	2	26.42	4.04	0.12	2.4
		Nonlinear	3	–	–	–	–
	Food (allowing for intra‐ and interspecific competition)	**Null**	**1**	**22.38**	**0**	**0.88**	**–**
		Linear	2	26.38	4.00	0.12	1.0
		Nonlinear	3	–	–	–	–
	Breeding habitat	**Nonlinear**	**3**	**13.24**	**0**	**0.99**	**94.5**
		Null	1	22.38	9.14	0.01	–
		Linear	2	25.44	12.20	0.00	15.0
Local	Breeding habitat	**Nonlinear**	**3**	**205.53**	**0**	**0.95**	**18.7**
		Linear	2	211.53	6.00	0.04	8.6
		Null	1	215.78	10.25	0.01	–

While the regional island archipelago populations have grown at similar rates, there is considerable variation in pgr across local populations in these regions, particularly when breeding habitat availability is low. Consistent with this greater variation, the most parsimonious model explained a lower percentage of the null model's deviance (19%) than occurred at the regional level. Nevertheless, the results support the conclusion that density‐dependent population growth in response to low breeding habitat availability also occurs at the local level (Table 1, Figure 4d). The local‐scale model predicts pgr declines below a maximum level of 2.3% per annum at <200 m^2^ of unoccupied habitat per breeding pair. The growth rates of local populations were the lowest at islands with small areas of breeding habitat (Figure 4d).

### 3.4 Occupancy of terrestrial habitat

**Figure 5:**
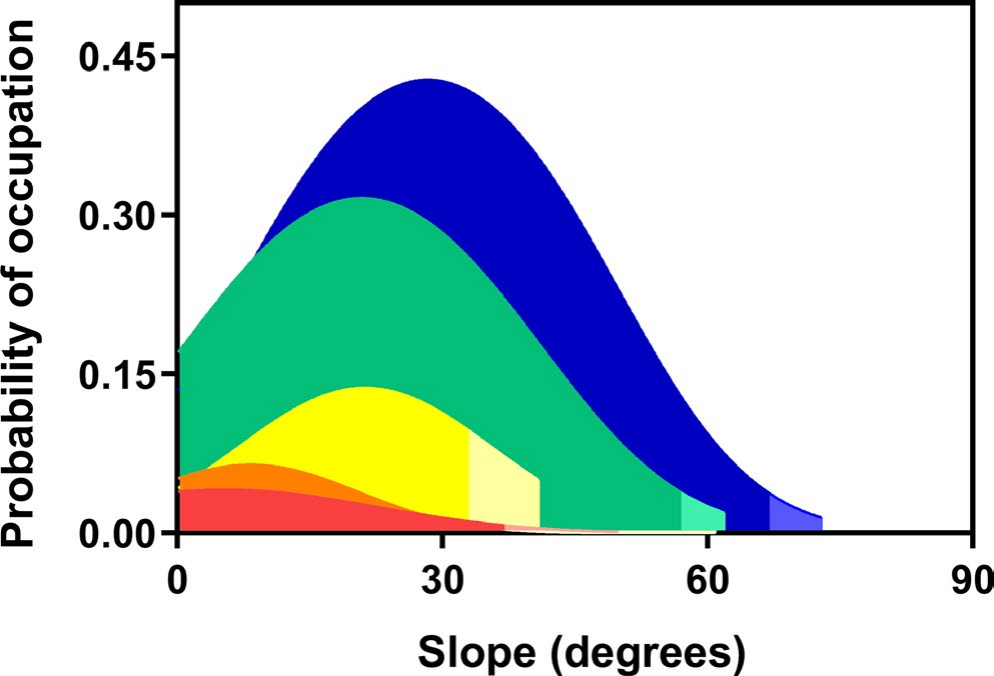
Predicted probability of Adélie penguins occupying ice‐free land in relation to slope for five regional populations with differing breeding habitat availabilities. island archipelago regions at the Windmill Islands, Vestfold – Rauer Islands, and Holme Bay Islands are red, orange, and yellow respectively; mountain nunatak regions at Mount Biscoe and Scullin–Murray Monoliths are green and blue. Breeding habitat availability decreases across this sequence of regions. Occupation probabilities are predicted across the full range of plot slopes present in each region. Curves are shaded to reflect the slopes occupied by penguins (dark) and predictions for other slopes that were available but unoccupied (pale)

The most parsimonious model for occupancy was the global model that included region, slope, slope^2^, and the interaction of region and slope (Table 2). This model characterizes the broad patterns of occupancy in relation to the regions and their physical environmental features but, because we did not account for spatial autocorrelation of occupancy related to the species colonial breeding behavior, explains a small percent of the null model's deviance (4%). The model predicts that occupancy is 2–7 times higher in mountain nunatak habitats than in island archipelago habitats (Figure 5). Region‐slope models predict that occupancy peaks at slopes in the range of 10–40° across the regions and that the peak occurs at greater slopes in mountain nunatak regions than in island archipelago regions (Figure 5). Penguins breeding in mountain nunatak habitats also occupy steeper slopes (>35°) that are sparsely occupied or not occupied by penguins in island archipelago habitats.

**Table 2:** Model selection results for a logistic generalized linear model of breeding habitat occupancy in relation to region and slope. Models are ranked in order of Akaike weights (*w_i_*), and those with substantial support (∆AIC < 2) are shown in bold

Candidate models for occupancy	*K*	AIC	∆AIC	*w_i_*	% of null model's deviance explained
**Region + Slope + Slope^2^ + Region:Slope**	**5**	**15,457**	**0**	**1.00**	**4.4**
Region + Slope^2^	3	15,644	187	0	3.8
Region + Slope + Slope^2^	4	15,646	189	0	3.8
Region + Slope	3	15,660	203	0	3.8
Region	2	15,731	274	0	3.4
Slope	2	16,275	818	0	0.1
Null	1	16,278	821	0	–

## 4 DISCUSSION

In this study, we exploit the natural spatial variation in marine and terrestrial environments of Adélie penguin breeding populations along the East Antarctic coastline to explore whether there is evidence for density‐dependent resource limitation constraining population growth. Against a history of strong positive Adélie penguin population growth across the breadth of East Antarctica (Southwell et al., 2015), two regional populations stand apart in showing little or no change. Both these populations, which are located in mountain nunatak terrestrial habitats, have relatively low breeding habitat availability, suggesting that their growth is likely constrained by density‐dependent limitation of breeding habitat. We also found evidence that growth of local populations was constrained at islands with limited breeding habitat despite strong positive growth over the last several decades in the regional populations in which they are located. We found no evidence that food availability has limited population growth, but our analysis is potentially limited by our forced use of proxies rather than direct data on food availability. The results of this study are a substantial development in Adélie penguin ecology because most recent studies have focussed on the importance of bottom‐up, extrinsic, marine processes for understanding Adélie penguin population dynamics (e.g., Cimino, Fraser, Irwin, & Oliver, 2013; Jenouvrier, Barbraud, & Weimerskirch, 2006; Smith et al., 1999), largely to the exclusion of intrinsic and terrestrial influences, as prediction of the effects from a changing environment has become more imperative and satellite‐derived data on the marine environment have become readily accessible. Here, we bridge this fundamental gap by assessing both intrinsic and extrinsic influences simultaneously to understand their differential roles in population limitation.

Our analysis of occupancy in relation to breeding habitat availability provides additional evidence in support of density‐dependent limitation and gives insight into the mechanism of how this could occur. Occupancy in mountain nunatak habitats is considerably higher than in island archipelago habitats, and Adélie penguins nest on steeper slopes in the mountain nunatak regions than at island archipelagos. We also observed Adélie penguins establishing nests further inland at Murray Monolith than in any of the other regions (up to 900 m; only up to 500 m elsewhere). Combined, these results are consistent with an ideal despotic distribution model where individuals search for high‐quality nest sites in a preemptive manner and subsequently occupy poorer habitat (in this case, steeper slopes and further from the coast) as population density increases (Fretwell & Lucas, 1969). While our study does not assess whether breeding success is lower in the steepest or most inland areas of the mountain nunatak regions, this is not a necessary consequence of the ideal despotic model as populations may simply cease to establish additional nests, and hence cease to grow, when the quality of breeding habitat reaches a critically low level. One possible consequence of higher occupancy rates in the mountain nunatak habitats is that these habitats are close to or at carrying capacity and may be a source of immigrants to other regions where breeding habitat is available. Other studies have highlighted the importance of source‐sink dynamics in Adélie penguin populations over yearly to millennial time‐scales (LaRue et al., 2013; Younger, Emmerson, Southwell, Lelliott, & Miller, 2015).

Given that Adélie penguin population growth is lacking in the mountain nunatak regions and large populations of breeding Antarctic petrels occur in those regions, it is worthwhile considering whether interactions between the two taxa could result in interference competition for breeding habitat. Interference competition for breeding habitat is known to differentially limit species' populations at seabird breeding assemblages elsewhere (Oro et al., 2009), but we think it is unlikely to be important in this case because of the differences in biology and nesting behavior of Adélie penguins and Antarctic petrels. Firstly, interference competition in seabird breeding assemblages is thought to be driven by a hierarchy of body size, whereby smaller species avoid breeding with larger species (Oro et al., 2009). In this case, size‐related interference competition would favor Adélie penguins over Antarctic petrels rather than vice versa. Secondly, the breeding habitat preferences of Adélie penguins and Antarctic petrels are strikingly different, the former favoring ice‐free land close to the coast and with low slope (Ainley, 2002), and the latter favoring cliffs and steep slopes (van Franeker et al., 1999; Schwaller, Lynch, Tarroux, & Prehn, 2018). Finally, Adélie penguins breed in dense colonies and commonly use collective aggressive behavior to defend against avian competitors or predators (Tenaza, 1971; Young, 2002), while Antarctic petrels are less aggressive in defense of nest sites (pers. obs).

In contrast to breeding habitat availability, we found no compelling evidence for food availability limiting regional population growth. The two mountain nunatak regional populations are key to demonstrating regional density dependence because of their low population growth rates compared with the other five regions. Low food availability in both mountain nunatak regions would likely indicate the presence of density dependence, but the indices for these regions were instead substantially different (Mount Biscoe at the lower end and Scullin–Murray Monoliths at the higher end of the food availability range).

The lack of evidence for food‐related density dependence could be explained in a number of ways. Most obviously, it could be that prey in the foraging range of the regional Adélie penguin breeding populations included in this study is superabundant relative to the size of penguin and seabird breeding populations. Dehnhard (2019), for example, propose that a lack of differentiation in the foraging locations of three sympatrically breeding fulmarine petrels in the Vestfold – Rauer region may be attributed to high productivity of food in that region. Alternatively, it could be that food is not superabundant and the potential for intra‐ and interspecific competition exists, but this potential is mediated by specific breeding and foraging strategies or responses. For example, Adélie penguins are thought to mediate intraspecific competition by geographic structuring of breeding colonies (Ainley, Nur, & Woehler, 1995) and by spatial partitioning of foraging in neighboring colonies (Ainley et al., 2004). Interspecific competition between Adélie penguins and flying seabird species is also likely to be mediated by their strong horizontal and vertical spatial partitioning in foraging (Clarke et al., 2006; Dehnhard et al., 2019; Descamps et al., 2016; Whitehead, 1989), even though they have broadly similar diets of krill and fish (Green & Johnstone, 1988; Lorensten, Klages, & Røv, 1998; Nicol, 1993; Tierney, Emmerson, & Hindell, 2009). A third explanation, given the notorious difficulty of quantifying competition and the abundance of mid‐trophic organisms in marine ecosystems (Oro, 2014), is that our indices did not accurately reflect the true availability of food. The indices have several caveats in this regard. Our use of primary productivity as an indirect proxy for food abundance is common practice in studies of marine predators, but empirical quantitative validation of the robustness of the proxy is limited. While polynyas are generally considered to be important features for foraging Adélie penguins, optimal foraging habitat may only occur in the marginal ice zone at the edge of polynyas (Lescroël, Ballard, Grémillet, Authier, & Ainley, 2014), or alternatively polynyas may provide easier access to more distant foraging grounds because their reduced sea‐ice allows more efficient travel (Emmerson, Walsh, & Southwell, 2019). Finally, although we accounted for potential intra‐ and interspecific competition from cohabiting breeding seabirds, other potential competitors that were not accounted for in the indices include nonbreeding individuals which can be as abundant as breeders (Southwell et al., 2017), seals that breed and forage in the Southern Ocean (Southwell et al., 2012), other seabird and marine mammal species that breed in more temperate locations but feed close to the Antarctic continent (Branch, 2011; DeLord et al., 2014, 2010; Raymond et al., 2015), and fish or squid species that predate on the same prey as penguins (Lyver et al., 2014). While improved knowledge of foraging locations of multiple species through technological advances and deployments (Wilmers et al., 2015) will go some way toward better conclusions on food‐related density dependence, the greatest progress in understanding food‐related density dependence will come with the development of new methods for estimating the abundance or biomass of mid‐trophic level organisms over large spatial scales (e.g., Ainley et al., 2015).

Our demonstration of density‐dependent population regulation for Adélie penguins in East Antarctic matches similar conclusions from studies in the Ross Sea and Antarctic Peninsula regions (Che‐Castaldo et al., 2017; Lyver et al., 2014), suggesting that density‐dependent processes may operate throughout the species' circumpolar distribution. The studies elsewhere reached their conclusions based on the finding of a negative relationship between population growth and population size. However, population growth will not necessarily reduce with increasing population size if resources are still abundant relative to a large population. Our approach to this issue differed by relating population growth to the per capita abundance of food and breeding resources rather than to population size. We suggest this approach is more direct and most likely to advance insights into the presence and drivers of density dependence in future studies.

In response to growing concern for how a future changing environment will affect biota worldwide (Walther et al., 2002), there has been a strong focus by ecologists to develop quantitative models to predict the future trajectory and state of species' distributions and populations, with several studies focussing on Antarctic penguins (Ainley et al., 2010; Ballerini, Tavecchia, Pezzo, Jenouvrier, & Olmastroni, 2015; Che‐Castaldo et al., 2017; Cimino, Lynch, Saba, & Oliver, 2016; Jenouvrier et al., 2009, 2012, 2014). Our study highlights the importance of incorporating nonconstant species–environment relationships in predictive models. Until recently, most species distribution and population dynamics models have implicitly assumed constant species–environment relationships by modeling relationships under present conditions and projecting the same relationships forward in time under changing environmental conditions (Elith & Leathwick, 2009). Our finding that Adélie penguins breeding in mountain nunatak habitats occur not only at higher occupancy rates, but also on steeper slopes and greater distances inland that are unoccupied in other regional populations, demonstrates that occupancy and habitat use are density‐dependent and can vary under certain conditions for this species. Nonconstant species–environment relationships may occur frequently in nature as populations decline or expand, and in the future, nonconstant relationships may also be driven by expanding or contracting habitats even if the populations using them are constant. A pertinent example of this scenario is the predicted increase in ice‐free land around the Antarctic continent as the climate warms in the future (Lee et al., 2017) coupled with the finding of altered population dynamics of Adélie penguins colonizing land recently exposed by glacial retreat (LaRue et al., 2013).

By demonstrating that processes operating in the terrestrial environment can constrain Adélie penguin population growth in some regions and local sites, our study highlights the potential for terrestrial processes to mask or confound marine influences on Adélie penguin population dynamics. This casts some caution on the often cited role of Adélie penguin populations as “sentinels” or “indicators” of fishery and climate change impacts on Southern Ocean marine environments (Agnew, 1997; Ainley, 2002), and suggests that a broadening of the indicator role may be required. However, despite this cautionary conclusion, our study offers some guidance to address this issue. The numerical response functions developed in this study predict the level of breeding habitat availability below which population growth is constrained, and hence where masking or confounding of marine influences has the potential to occur. If population monitoring is only possible at a small number of local breeding sites, focussing at sites that exceed this predicted level of breeding habitat availability would minimize any potential masking by terrestrial influences, and also increase the ability to detect change by reducing the inherent variation in the monitored system. Alternately or additionally, monitoring at multiple sites to estimate regional‐scale population change would avoid overestimating the importance of local‐scale processes in regional‐scale dynamics. Our results highlight the importance of understanding constraints for multiple populations in similar and varying environments. One of the challenges in the future will be to design monitoring studies that are sufficiently adaptive for effective management under a changing environment and with increasing human pressures that could sway the balance between impacts in terrestrial and marine environments.

## CONFLICT OF INTEREST

There are no competing interests related to this work.

## AUTHOR CONTRIBUTIONS

Both authors contributed to all stages of the work.

## Data Availability

Data will be archived and available through the Australian Antarctic Division Data Centre (AADC) subject to a 12‐month embargo period after manuscript publication.
