# Supplementary material for: Density dependence forces divergent population growth rates and alters occupancy patterns of a central place foraging Antarctic seabird
Source: Ecol Evol. 2020 Feb 20;10(5):2339–51. doi: 10.1002/ece3.6037 (PMC7069296; doi:10.1002/ece3.6037)
Supplement: Supplementary file 1 [file ECE3-10-2339-s001.docx]

Appendix 1. Details of published Adélie penguin population count methods and population estimates at Mount Biscoe, and commentary on their contribution to estimating population growth rate in this study.

| **Breeding season** | **Survey and estimation details** | |
| --- | --- | --- |
| 1985/86 | Reference | Bassett et al. (1990) |
|  | Count details | Counts of individual adults by observers on the ground on 27-29 October 1985, but these counts were considered by the authors to be unsuitable and were not used for population estimation because they were made very early in the breeding season before most penguins had arrived. The authors state that counts later in the breeding season were needed to obtain an accurate estimate of population size |
|  | Details of published population estimation method | The published population estimate is based on the extent of guano, but no details are given and it is a minimum-only estimate with no upper bound. |
|  | Published population estimate | > 5,000 breeding pairs |
| 2010/11 | Reference | Lynch and LaRue (2014) |
|  | Count details | Estimate of guano area from satellite imagery obtained on 15 February 2011 |
|  | Details of published population estimation method | Guano area converted to population estimate from established relationship between guano area and population size at a sample of sites in the Ross Sea and Antarctic Peninsula |
|  | Published population estimate | 28,536 (17,415 – 47,225) breeding pairs |

**Commentary**

We considered there were insufficient population count data to estimate population growth because the baseline estimate in 1985/86 was a minimum-only estimate and hence not reliable for this purpose.

**References**

Bassett, J.A., Woehler, E.J., Ensor, P.H., Kerry, K.R., Johnstone, G.W. (1990) Adelie penguins and Antarctic petrels at Mount Biscoe, western Enderby Land, Antarctica. Emu 90: 58-60.

Lynch HJ, LaRue MA (2014) First global census of the Adélie penguin. The Auk 131: 457-466.

Appendix 2. Details of estimates of Adélie penguin guano area at Mount Biscoe, and commentary on their contribution to estimating population growth rate in this study.

| **Breeding season** | **Estimation details** | |
| --- | --- | --- |
| 1985/86 | Source | Fig. 1 in Bassett et al. (1990) |
|  | Estimation details | A copy of Fig. 1 from Bassett et al. (1990) and a WorldView2 high-resolution satellite image obtained on 15 February 2011 which included Mount Biscoe were imported into a GIS. The figure was geo-referenced to prominent rock features in the satellite image. The boundaries of guano shown in the figure were digitised as a set of polygon shapefiles and the area of the polygon files was measured. |
|  | Estimate of guano area (m^2^) | 167,724 |
| 2002/03 | Source | Fig. 2 and Supplementary Data in Schwaller et al. (2013) |
|  | Estimation details | The area of guano was calculated by multiplying the number of 30x30 m Landsat pixels at Mount Biscoe with a *d*-value of ≤0.50 (*n* = 211) by the area of each pixel (900 m^2^). Pixels with *d* ≤0.50 were considered by Schwaller et al. (2013) to have a high ‘goodness’ classification for Adélie penguin guano. |
|  | Estimate of guano area (m^2^) | 189,900 |
| 2010/11 | Source | Lynch and LaRue (2014) |
|  | Estimation details | The area of guano was calculated from high-resolution (0.6 m) Digital-globe satellite imagery obtained on 15 February 2011. Guano boundaries were delineating by hand or automatically using supervised classification and a subsequent maximum likelihood classification. |
|  | Estimate of guano area (m^2^) | 42,054 |

**Commentary**

At face value, it appears there has been a marked reduction in guano area in recent years (ie from 2002/03 to 2010/11). However, these areas were derived from satellite imagery with markedly different resolutions (30m and 0.6 m respectively) and are unlikely to be comparable. For this study, we used the guano areas from 1985/86 and 2002/03 to estimate population growth. These are both relatively coarse measures but are more comparable and therefore more suitable for inferring population change.

**References**

Bassett, J.A., Woehler, E.J., Ensor, P.H., Kerry, K.R., Johnstone, G.W. (1990) Adelie penguins and Antarctic petrels at Mount Biscoe, western Enderby Land, Antarctica. Emu 90: 58-60.

Lynch HJ, LaRue MA (2014) First global census of the Adélie penguin. The Auk 131: 457-466.

Schwaller, M.R., Southwell, C.J. and Emmerson, L.M. (2013) Continental-scale mapping of Adélie penguin colonies from Landsat imagery. *Remote Sensing of Environment* 139: 353-364.

Appendix 3. Average annual percentage population growth rate (*pgr*) of seven regional Adélie penguin breeding populations across East Antarctica over recent decades. Details of how *pgr* was estimated are provided in the sources given in the table. Briefly, *pgr* was estimated by fitting a linear regression of the natural logarithm of standardised population size estimates or indices against year and taking the slope as an estimate of *pgr* across the span of the time series. POP: Standardised population estimate; GUA: Guano area. * Population growth rate estimates presented in Southwell and Emmerson (2019) were derived from data including the most recent counts in 2017/18, but for this study population growth rate was calculated up to 2010/11 only to standardise the time period as closely as possible to the other regions.

| Regional population | | Population metric | Time period  (duration in years, number of data points in time series) | *pgr*  (median and 95 percentile range from bootstrapping) | Source |
| --- | --- | --- | --- | --- | --- |
| 1 | Lützow-Holm Bay | POP | 1981/82 to 2009/10 (28, 21) | 2.45 (1.90 to 3.04) | Southwell et al. (2015) |
| 2 | Mount Biscoe | GUA | 1985/86 to 2002/03 (17, 2) | 0.78 | This study |
| 3 | Holme Bay | POP | 1981/82 to 2009/10 (28, 4) | 2.27 (1.09 to 4.45) | Southwell et al. (2015) |
| 4 | Monoliths* | POP/GUA | 1986/87 to 2010/11 (24, 3) | -0.01 (-1.17 to 1.28) | Southwell and Emmerson (2019) |
| 5 | Vestfold-Rauer Islands | POP | 1981/82 to 2009/10 (28, 4) | 2.28 (1.62 to 3.02) | Southwell et al. (2015) |
| 6 | Windmill Islands | POP | 1972/73 to 2009/10 (37, 4) | 1.98 (1.51 to 2.51) | Southwell et al. (2015) |
| 7 | Terre Adélie Islands | POP | 1984/85 to 2009/10 (25, 4) | 1.78 (1.62 to 1.94) | Southwell et al. (2015) |

**References**

Southwell, C., L. Emmerson, J. McKinlay, K. Newbery, A. Takahashi, A. Kato, C. Barbraud, K. Delord, and H. Weimerskirch. (2015). Spatially extensive standardized surveys reveal widespread, multi-decadal increase in East Antarctic Adelie penguin populations. PLoS ONE **10**:e0139877.

Southwell, C., L. Emmerson (2019). Constraint in the midst of growth: decadal-scale Adélie penguin (*Pygoscelis adeliae*) population change at Scullin and Murray Monoliths diverge from increases elsewhere across East Antarctica. Polar Biology 342: 1397-1403.
